# Active Listening for Spatial Orientation in a Complex Auditory Scene

**DOI:** 10.1371/journal.pbio.0040079

**Published:** 2006-03-07

**Authors:** Cynthia F Moss, Kari Bohn, Hannah Gilkenson, Annemarie Surlykke

**Affiliations:** **1**Department of Psychology, Institute for Systems Research, University of Maryland, College Park, Maryland, United States of America; **2**Institute of Biology, University of Southern Denmark, Odense, Denmark; University of TexasUnited States of America

## Abstract

To successfully negotiate a complex environment, an animal must control the timing of motor behaviors in coordination with dynamic sensory information. Here, we report on adaptive temporal control of vocal–motor behavior in an echolocating bat, *Eptesicus fuscus,* as it captured tethered insects close to background vegetation. Recordings of the bat's sonar vocalizations were synchronized with high-speed video images that were used to reconstruct the bat's three-dimensional flight path and the positions of target and vegetation. When the bat encountered the difficult task of taking insects as close as 10–20 cm from the vegetation, its behavior changed significantly from that under open room conditions. Its success rate decreased by about 50%, its time to initiate interception increased by a factor of ten, and its high repetition rate “terminal buzz” decreased in duration by a factor of three. Under all conditions, the bat produced prominent sonar “strobe groups,” clusters of echolocation pulses with stable intervals. In the final stages of insect capture, the bat produced strobe groups at a higher incidence when the insect was positioned near clutter. Strobe groups occurred at all phases of the wingbeat (and inferred respiration) cycle, challenging the hypothesis of strict synchronization between respiration and sound production in echolocating bats. The results of this study provide a clear demonstration of temporal vocal–motor control that directly impacts the signals used for perception.

## Abstract 2

Recordings of sonar vocalizations synchronized to high speed video images reveal adaptive changes in vocal-motor behavior of echolocating bats as they catch insects in varying vegetation.

## Introduction

Echolocating bats rely on active sensing through acoustic channels and can orient in complete darkness. They produce ultrasonic vocal signals and use information contained in the returning echoes to determine the direction and distance of objects in space (reviewed in [ 1]). With their biological sonar, bats can successfully forage and avoid obstacles by rapidly processing spatial information carried by echoes of their sonar broadcasts. In acoustically complex environments, where many objects are present, the bat must organize echo information received from multiple sonar targets arriving from different directions and at different times. The bat's perceptual processes and motor control must operate in concert to enable auditory scene analysis and spatial orientation by sonar in a complex environment [ 2]. Echolocation depends on the dynamic interplay between vocal production and auditory processing [ 3]. Like active vision, which involves the coordination of eye, head, and body movements with the processing and interpretation of retinal images (see [ 4– 6]), echolocation gives rise to spatial perception from neural computations within and across both sensory and motor systems.

In this study, we exploit the active sensing system of the echolocating bat, *Eptesicus fuscus,* to gain direct access to the acoustic signals that drive the animal's behavior as it orients in a complex environment. *E. fuscus,* a species that relies on frequency-modulated (FM) signals to hunt for insect prey, is generally considered an open-space forager [ 7]; however, more recent reports indicate that this species also captures insects near the ground and vegetation [ 8], suggesting a wider repertoire of sonar-guided behavior than previously documented.

As E. fuscus flies toward an insect prey item, the features of its sonar vocalizations change, and the dynamic sonar sound characteristics reflect adaptive signal designs for different acoustic tasks: target detection, localization, and tracking [ 3, 9]. Comparatively long duration (up to 25 ms in the open field), shallow FM signals produced during the search phase are adapted for target detection, as sound energy is concentrated in a narrow frequency band over an extended period of time; shorter duration (0.5–6 ms), broadband approach, and terminal phase signals are adapted for target localization in azimuth, elevation, and range [ 1, 10]. Over the course of insect capture, the bat actively adjusts the duration and repetition rate of the signals to avoid overlap of sonar emissions and echoes [ 11, 12].

Previous studies of the sonar behavior of FM bats pursuing targets close to clutter have focused on the acoustic characteristics of the single pulse. The bandwidth increases and the pulse duration decreases in the presence of echo clutter (e.g. [ 13]). An increase in the signal bandwidth and rate of frequency modulation of single sounds can sharpen the bat's spatial resolution of objects in space. However, it is important to consider that bat echolocation is an adaptive perceptual modality with access to streams of dynamic acoustic information, and bats respond to sonar task demands not only by adjusting individual sonar cries, but also by modifying temporal patterns across many signals. Although past research reports on bat echolocation behavior have noted the increase in repetition rate with decreasing target distance [ 14], details of the temporal patterning layered over this general trend have been largely overlooked.

In the present study, we focus on the observation that bats produce and process sounds in sequences, which may carry information above and beyond that available from single cries. We hypothesize that cluttered conditions will evoke adjustments in the bat's temporal patterning of sonar call sequences, which impact the acoustic information available for goal-directed behavior (target capture) and obstacle avoidance. Specifically, we hypothesize that the bat actively produces and controls the temporal parameters of “sonar strobe groups” when it encounters spatially challenging acoustic tasks. Sonar strobe groups contain sequences of clustered pulses that are embedded in the search and approach phases of insect capture. When the sound groups contain three or more sounds, the intervals within the group are relatively stable (less than 5% deviation from the mean pulse interval [PI] within a group). The constant PI within strobe groups over long periods of time may be used by the bat to sharpen the spatial display of its environment. Consistent with the hypothesis that complex acoustic environments evoke the bat's production of sonar strobe groups are data showing that temporal patterning of acoustic signals can selectively shape the response profiles of echo delay-tuned neurons in the bat auditory system [ 15– 17]. An extension of the hypothesis that the bat controls the temporal patterning of its cries in response to sonar task demands is the suggestion that the temporal patterning of sonar cries and returning echoes may serve to modulate activity in populations of neurons that encode spatial information from the environment.

We predict that an insect positioned close to clutter will drive the bat to decrease the temporal separation of signals within strobe groups during its approach to a target, allowing more samples of the environment per unit time. Furthermore, we predict that clutter will reduce the time period over which the bat produces the terminal buzz [ 2], as background vegetation creates a cascade of echoes following each sonar pulse, and extended echoes compromise the buzz's utility for high sampling of the environment just before target capture. Here, we analyze these vocal parameters in the context of flight path control, which the bat can use to enhance directional separation of information carried by echo returns from the target and background vegetation ( Figure 1). Our results reveal adaptive motor behaviors that support spatial orientation in a complex auditory scene.

**Figure 1:**
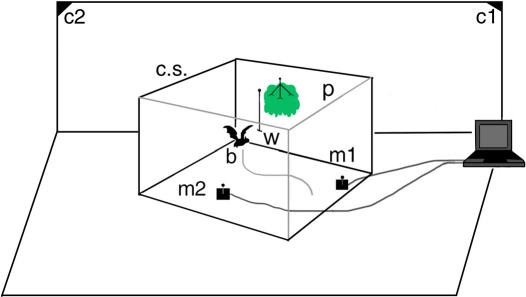
Schematic of Flight Room Shows setup for video and sound recordings of flight path and acoustic behavior of bats (b) capturing tethered mealworms (w) close to an echo clutter-producing plant (p). Two high-speed video cameras (c1 and c2) were mounted in the room to permit 3-D reconstruction of the bat's flight path in the calibrated space (c.s.). Video recordings were synchronized with audio recordings taken with ultrasonic microphones (m1 and m2) placed on the floor delivering signals to a digital acquisition system (IOtech WaveBook).

## Results

With the tethered insect as close as 10 cm from the plant, echolocating bats made no attempt to capture the target in nearly 50% of all trials; the bats successfully hit or captured the insect in about 40% of the trials, whereas approximately 10% resulted in aborts or misses. The percentage of attempts and successful captures increased dramatically as the target–clutter separation increased. When the tethered worm was positioned 20 cm from the clutter, target hits/captures increased to almost 80%, and trials in which the bat made no attempt to capture the insect dropped below 10%. When the target was positioned 40 cm from the clutter, 90% of the trials resulted in hits/captures of the target, and the bat made an attempt to intercept the insect on every trial. Finally, in the open room the bat captured or hit the worm in nearly all trials, and there were no trials without attempts or resulting in aborts or misses. Differences in performance across conditions are statistically significant following an analysis of variance (ANOVA; *F* = 29.45, *p* < 0.001), and the data are summarized in Figure 2.

**Figure 2:**
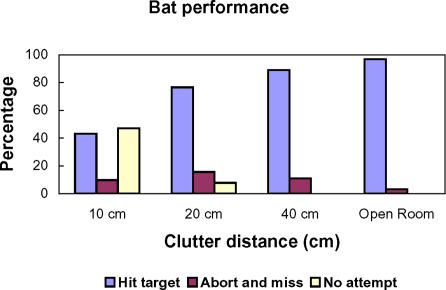
Bat Performance Percentage of attempts and successful captures increased as the target–clutter separation increased, whereas the percentage of trials in which animals made no attempt to capture the target decreased. The bats' performance was tested in 51, 63, 70, and 32 trials at clutter distances of 10, 20, and 40 cm and open room, respectively.

Before attempting capture of insects close to clutter, the bat sometimes flew around the room for many tens of seconds. The further the insect was from the branches, the more quickly the bat moved in to intercept its prey. The large and significant decrease in the trial duration with the increase in target–clutter separation ( *F* = 26.63, *p* < 0.001) is shown in Figure 3.

**Figure 3:**
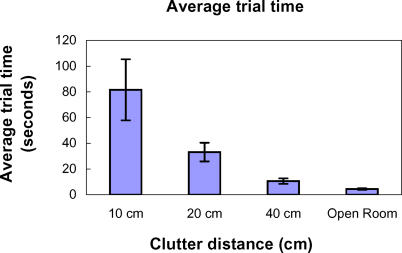
Average Trial Time The trial duration decreased with increasing target–clutter separation to the shortest duration for open room, measured in 11, 27, 36, and 31 trials at 10-, 20-, and 40-cm clutter distance and open room, respectively.

Recordings from the plant and worm reveal how weak the target echo is compared with that of the plant. The target strength of the worm is about −24 dB, while the plant is, on average, −15 dB. How then, does the bat manage to detect, localize, and intercept the tethered worm when it is positioned 20 cm or closer to the plant? The bat's flight path and vocal behavior may provide some insights to the motor strategies employed by bats faced with this difficult echolocation task. Therefore we reconstructed the bat's three-dimensional (3-D) flight paths and plotted them along with the vocal temporal patterns recorded from selected trials run with target–clutter separations of 10, 20, and 40 cm and the open room (see Figure 4). Animations for these trials are available at http://www.bsos.umd.edu/batlab/3Danimation.html.

**Figure 4:**
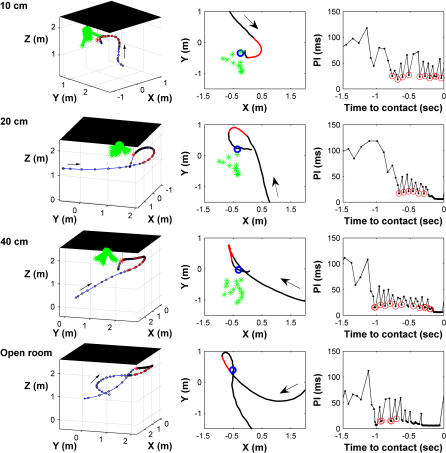
Bat Flight Paths Shows plots of the bat flight paths and vocal temporal patterns recorded from three selected trials run with target–clutter separations of 10, 20, and 40 cm and open room. The far-left side shows 3-D plots of the bat's flight path with respect to the plant. The direction of the bat's approach is indicated by an arrow. The middle section shows overhead perspectives on the same four trials. Positions of branches are indicated by green asterisks and position of the worm is indicated by blue circles. The segment of the flight path shown in red corresponds to the time period in which the bat produced strobe groups. Again, arrows indicate the direction of the bat's flight path. The far-right side plots PIs of the sonar signals recorded during the approach and terminal phases of insect pursuit in each trial. Note that the example at 10 cm shows no buzz, as this was an aborted trial. In the open room example, the bat produced strobe groups as it first flew close to the target, but not in the final approach and interception of the target. Each of these examples shows a decrease in PI as the bat approaches the target and clear examples of sonar strobe groups. The strobe groups are characterized by stable PIs (up to 5% variation about the mean PI), interrupted by breaks that are at least 1.2 times the mean PI. The strobe groups produced by the bat in each of these examples are circled in the PI plots. In many instances, the production of strobe groups occurs over hundreds of milliseconds.

Figure 4 also shows overhead plots of the bat's flight path with respect to the plant for trials with target–clutter separations of 10, 20, and 40 cm and the open room. Asterisks show the position of selected branches of the plant. Note that the bat did not fly directly to the insect prey but instead flew alongside the plant and the tethered worm, before turning in for a capture. This implies that for some portion of each trial, the bat minimized backward masking of the insect echo by the plant clutter echo by ensonifying the prey target off axis from the plant. The trials illustrate typical flight path patterns for the different clutter conditions: the closer the clutter the more abrupt is the final turn and the shorter the distance (and time) traveled directly toward the target. In these plots, the segment of the flight path in which the bat produces strobe groups occurs in red. Sonar strobe groups are sound clusters bracketed by longer intervals (>1.2 times the mean interval within the sound group) to neighboring strobe groups or signals. For strobe groups containing three or more signals, the PI of each group is stable with a tolerance of 5%. The occurrences of sonar strobe groups are shown in the PI plot of Figure 4 (circled in red) for the final 1.5 s before target contact. Note that as the bat approaches the target the overall PI decreases; however, the decrease is not monotonic in the individual trial, and long periods of sonar strobe group production are evident.

Time waveforms and spectrograms of sounds produced by bats in these example trials are presented in Figure 5 to further illustrate the sonar strobing phenomenon. This figure shows vocalization data from single trials under each of the four conditions: 10-, 20-, 40-cm clutter separation and open room. The far-left side plots the time waveforms from approximately 2.5 s before target contact to 0.5 s after target contact. The region shaded in pink displays the portion of each trial that is presented in the PI plots of Figure 4, 1.5 s before the time of target contact. Spectrograms of the trial segment shaded in gray are presented in Figure 5 on the far-right side. The sounds that are defined as belonging to sonar strobe groups are identified with open red circles marked along the time axis below the spectrograms. Along this time axis, blue circles mark the occurrence of all other sounds. Animations for the data are available at http://www.bsos.umd.edu/batlab/strobing.html.

**Figure 5:**
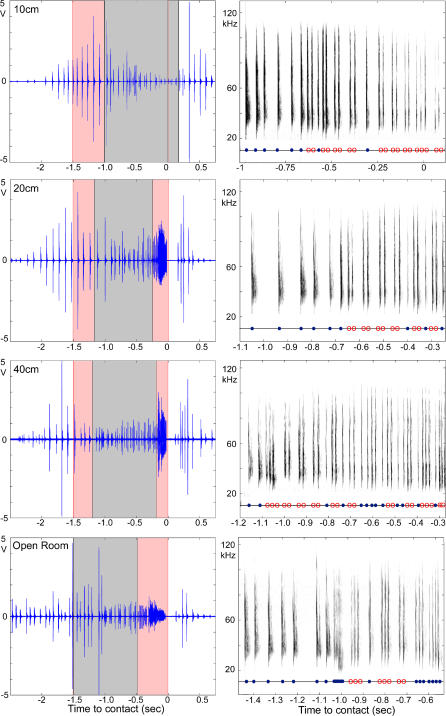
Time Waveforms and Spectrograms Displays time waveforms and spectrograms of the sounds produced in the four trials presented in Figure 4 for 10-, 20-, and 40-cm clutter separation and open room. The left side plots the time waveforms from approximately 2.5 s before target contact to 0.5 s after target contact. The shaded regions display the portion of each trial that is presented in the PI plots of Figure 4, starting at 1.5 s before the time of target contact. Spectrograms of the trial segment shaded in gray are presented in Figure 5 on the right side. The sounds that are defined as belonging to sonar strobe groups are identified with open red circles marked along the time axis below the spectrograms. Along this time axis, blue filled circles mark the occurrence of all other sounds.

Wingbeat cycle is tied to respiration in the bat, with inhalation occurring during the downstroke and exhalation during the upstroke [ 18]. Breaks in the sound groups would be expected to occur during a particular phase of the downstroke if wingbeat and respiration were strictly driving the production of sound groups. Figure 6A shows the timing of sounds from a selected open room trial and the relation of the sonar strobe groups to the bat's wingbeat cycle, measured from the high-speed video recordings. Wingbeat rate was on average about 12 Hz and did not change dramatically during approach to prey and clutter. This plot shows, however, that the sound groups occur across all phases of the wingbeat cycle and certainly through the entire terminal buzz, suggesting that the bat's vocal control can override the wingbeat/respiration cycle [ 2]. The bat exercises control over the PIs within and between sounds in strobe groups, which depend on clutter conditions and time-to-contact.

**Figure 6:**
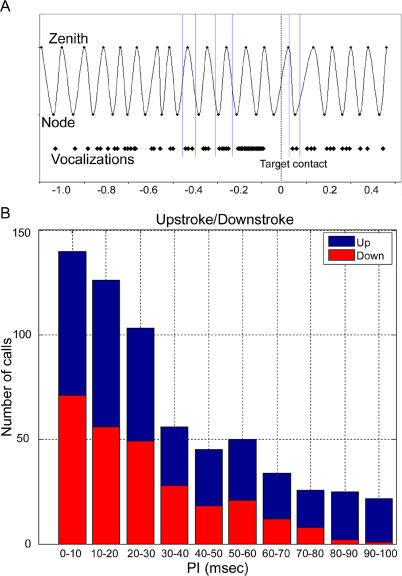
Control over Timing of Pulse Emission (A) shows the timing of sounds from a selected trial and the relation of the sonar strobe groups to the bat's wingbeat cycle, measured from the high-speed video recordings and illustrates that the coupling to wingbeat rate is not strict. (B) plots the distribution of sounds during the upstroke (exhalation; shown in blue) and downstroke (inhalation; shown in red) of the bat's wingbeat for sounds with different PIs, over nine trials. The total number of vocalizations included in the 10-ms PI time bins increases with decreasing PI, because the bat increases the number of vocalizations at shorter intervals as it approaches the target. Note that the distribution of sounds produced during the upstroke and downstroke of the wingbeat cycle becomes more similar for PIs shorter than 50–60 ms (approximately 17–20 sounds/s).

Figure 6B plots the temporal distribution of sound production referenced to the upstroke (shown in blue) and downstroke (shown in red) of the wingbeat cycle as the bat approaches insect prey in nine separate trials. The number of samples included in each time bin increases with shorter PIs, as the bat produces more sounds at shorter intervals as it approaches a target. When the PI is greater than 80 ms, the bat tends to produce sounds predominantly during the upstroke of the wingbeat cycle; however, for shorter PIs, the number of sonar sounds occurring during the downstroke of the wingbeat cycle tends to increase. For PIs less than 50–60 ms, the sounds occur equally frequently during the up- and downstrokes of the wingbeat cycle.

In the final approach to the target before interception, the bat's turning rate increases under clutter conditions compared with the open room ( Figure 7). The increase in turning rate is most dramatic in the final 50–100 ms before contact and appears very late with respect to time of contact for the 10-cm target–clutter separation; by contrast, under the open room condition the approach is a shallow curve where there appears to be little change in turning rate. An ANOVA on the turning rate data reveals a statistically significant difference in turn rate between clutter and open room conditions over the final 100 ms before target capture ( *F* = 4.78, *p* < 0.05) but no statistically significant difference across conditions in the period between 100 and 700 ms before capture ( *F* = 1.08, *p* > 0.1).

**Figure 7:**
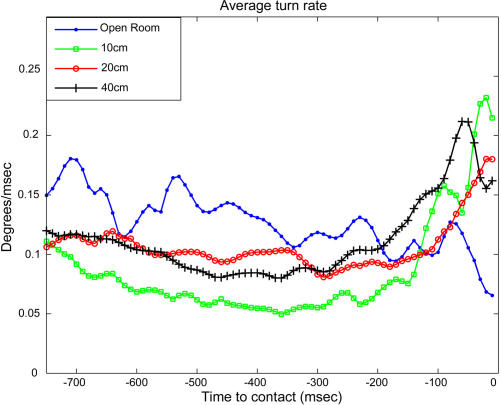
Average Turn Rate Plots mean turning rate of the flying bats in the three clutter conditions (10 cm, *n* = 7; 20 cm, *n* = 23; 40 cm, *n* = 29 trials included in the analysis) and the open room ( *n* = 10 trials), referenced to target contact time (zero on the abscissa). There was a significant difference in the bat's turning rate between clutter and open room conditions in the final 100 ms before target contact ( *F* = 4.78, *p* < 0.05).

The flight path adjustments during the final attack on the insect also coincided with a decrease in the terminal buzz duration with decreasing target–clutter separation. The mean duration of the buzz that preceded capture attempts in the open room and for each of the three target–clutter separations is presented in Figure 8. Only buzz II (PIs of less than 8 ms; [ 10]) was included in this analysis. Buzz duration dropped significantly with target–clutter separation, from over 300 ms in the open room to about 200 ms when the tethered prey was 20–40 cm from the clutter, down to 100 ms when the target was only 10 cm from the clutter (ANOVA; *F* = 24.28, *p* < 0.001). Bats attempted to take the tethered insect at a much lower rate when the target was positioned 10 cm from the clutter (see Figure 2), so the buzz data reported here for this condition are less reliable. The increased turn rate under conditions of clutter only appeared in the final 100 ms before target capture, after the buzz onset. The observation that the mean buzz onset time occurred before the bat's average turning rate increased suggests that the two behaviors are not coupled; however, more complete consideration of their possible relation requires further study.

**Figure 8:**
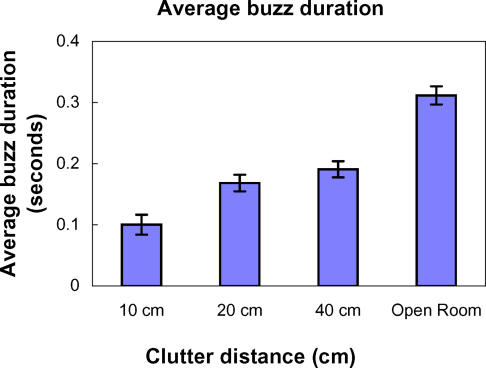
Average Buzz Duration Plots the mean duration of the buzzes that preceded capture attempts in the open room ( *n* = 27) and for each of the three target–clutter separations: 10 cm ( *n* = 2), 20 cm ( *n* = 23), and 40 cm ( *n* = 32); *n* is number of buzzes recorded in trials with good signal-to-noise-ratio sound recordings. Only buzz II (PIs of less than 8 ms [ 10]) was included in this analysis.

Figure 9 summarizes average signal parameters measured in 100-ms time intervals before capture in the open room and for each of the clutter conditions (10, 20 and 40 cm target–clutter separations). ANOVAs were conducted separately for each signal parameter to determine if differences in vocalizations were statistically reliable across conditions, and Bonferroni corrections were taken to adjust the α levels for the multiple tests. (Attempts at testing all the data in a multivariate analysis of variance failed to converge.) Each ANOVA used time (to target contact) in a repeated-measure model, with clutter condition (10, 20, 40 cm, and open room) as the independent variable. Not surprisingly, there was a significant effect of time for all parameters tested ( *p* < 0.001). Surprisingly, however, there was no main effect of condition on signal duration ( *F* = 0.98, *p* = 0.4). The only signal parameter that showed a statistically significant main effect across open room and clutter conditions was the PI ( *F* = 12.41, *p* < 0.01). There was also a statistically significant interaction between time to contact and clutter condition for the end frequency of the sweep ( *F* = 2.57, *p* < 0.05). There was a marginally significant interaction between time and condition for sweep rate ( *F* = 1.75, *p* < 0.07). The results of these analyses point to the importance of timing of sound production as the bat responds to the acoustic clutter.

**Figure 9:**
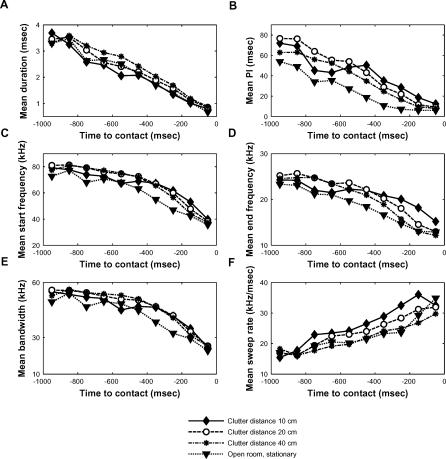
Signal Parameters Summarizes average signal parameters measured in 100-ms time intervals before capture in the open room and for each of the clutter conditions: 10-cm ( *n* = 7), 20-cm ( *n* = 25), and 40-cm ( *n* = 34) target–clutter separations and open room ( *n* = 11); *n* is number of trials included in the analyses. Those included had good signal-to-noise ratios, permitting reliable analysis of bat sonar cries throughout the trial. (A) and (B) show temporal parameters (duration and PI), and (C–E) show spectral parameters, start frequency, end frequency, and bandwidth of the fundamental. (F) shows sweep rate. There was a statistically significant main effect of clutter condition on PI ( *F* = 12.41, *p* < 0.001) but not on other sound parameters (see text for details on the ANOVA).

Data from the individual trials demonstrate that the bat produces sonar strobe groups during the approach phase of insect capture (see Figures 4 and 5), a finding that has received little attention in the bat echolocation literature. Strobe groups were produced by the bat under all clutter conditions and in the open room, but the incidence was higher under clutter conditions during the final approach to the insect. Figure 10A summarizes this finding, with mean percentage of time strobing before target contact. Figure 10B plots the PI of sounds produced by a bat in a single trial to illustrate the occurrence of sonar strobe groups. Figure 10A illustrates that the percentage of time strobing was consistently high in the period before capture for the 20-cm clutter condition, whereas strobing increased only in the final 300–500 ms before contact in the 40-cm clutter condition. Statistical tests show that the difference in percentage of time strobing is reliable between clutter and open room conditions in the time interval between 300 and 500 ms before contact ( *F* = 5.2, *p* < 0.05), but not between the two different clutter conditions (20- and 40-cm target–clutter separations; *F* = 2.76, *p* > 0.1). In the time bin at 900 ms before contact, the pattern was different: there was no statistical difference between the percentage of time strobing in the open room and 40-cm clutter condition ( *F* = 2.35, *p* > 0.1); however, strobing occurred at a statistically higher incidence under the 20-cm clutter condition ( *F* = 4.7, *p* < 0.05). Note that the percentage of time strobing was zero in all conditions at the −100-ms time bin (average of data taken between −200 ms and target contact), as this occurred during the terminal buzz, which was excluded from the strobing analysis. The bat approached the target in only a few trials when the target was placed 10 cm from the clutter, and therefore data from this target–clutter separation were not included in this analysis.

**Figure 10:**
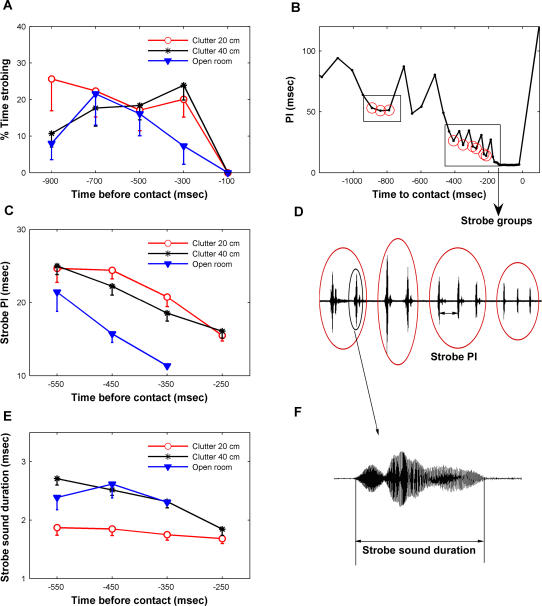
Sonar Strobing Behavior Summarizes the analysis of sonar strobing behavior (see text for definition) in bats attempting insect capture under different clutter conditions. Included in the analyses summarized in (A), (C), and (E) are data from trials in which the bat hit or captured the insect positioned 20 cm from the clutter (17 trials), 40 cm from the clutter (25 trials), and the open room (11 trials). There were too few successful trials at 10 cm to include in these analyses. Note that the time axes differ across plots in this figure. (A) Shows the mean percentage of time the bats produced sonar strobe groups during the 1,000-ms time period before target contact. Data points plot the mean percentage time strobing at midpoints of 200-ms intervals (e.g., data point at −300 ms shows the mean percentage time strobing between the time interval 200–400 ms before target contact). Note that the highest incidence of strobing at 900 ms or more before contact occurred when the bat encountered the target 20 cm from the clutter. (B) Plots the PIs of successive sounds taken from a single trial, showing changes that occur in the temporal patterning of vocalizations before target contact. The strobe groups are circled in red. (C) Plots the mean PIs of sounds contained within strobe groups under open room and clutter conditions, averaged across 100-ms time intervals during the time period 600–200 ms before target contact. For example, the data point at −350 ms shows the mean strobe PI between −300 and −400 ms. (D) Time waveforms of sonar strobe groups taken from the data shown in (B) are displayed. The strobe groups are circled. Measurement of strobe PI is indicated in one of these strobe groups. (E) Plots the mean duration of sounds contained in strobe groups for the clutter and open room conditions, again referenced to target contact time and averaged over 100-ms time bins. (F) Illustrates the measurement of strobe sound duration for one of the sounds in the strobe groups shown above in (D).

Figure 10C plots the mean PIs of sounds within strobe groups during the final approach to the insect target. Measurement of strobe PI is illustrated in Figure 10D. Data in 10C are presented separately for the open room and clutter conditions (20- and 40-cm target–clutter separations). Note that the PI within the sonar strobe groups increases with decreasing clutter distance: the difference in PI across conditions is statistically significant ( *F* = 4.74, *p* < 0.05). Figure 10E plots the duration of sounds in the corresponding strobe groups for clutter and open room conditions. Measurement of sonar sound duration is illustrated in Figure 10F. The duration of sounds in the strobe groups produced by the bat before target contact is shorter for the closest target–clutter separation (20 cm). Sound duration differences between open room and 40-cm clutter is not statistically significant, but differs reliably from the sound duration of strobe group sounds produced in the 20-cm clutter condition ( *F* = 17.44, *p* < 0.001). Note that the sound duration data included in this analysis are a subset of those included in (A) of Figure 9, and therefore differences in the results of statistical tests presented in Figure 10 and above are not contradictory.

## Discussion

Echolocating bats exhibit dynamic sound production patterns that reflect adaptive responses to changing acoustic information from objects in the environment. Indeed, the bat's adaptive vocal production patterns provide a window to the information sought and collected by the active sonar system for acoustic imaging under different task conditions. In the present study, we report that the bat reduces the interval between successive sounds as it approaches prey, but the decrease is not monotonic within single trials. In particular, the bat produces “sonar strobe groups” during the approach phase, in which the signals are produced in clusters and PI is stable (5% tolerance), sometimes for many hundreds of milliseconds ( Figures 4, 5, and 10). These adaptive vocal patterns shape the information that contributes directly to the bat's spatial representation of the auditory scene and facilitate spatial orientation in the environment [ 2].

Action and perception are intimately linked, and examples of their coupling occur in a variety of modalities and different animal species, including the role of eye movements and locomotion on visual perception in primates [ 19], whisking behavior on tactile sensing in rodents [ 20], and electric organ discharge patterns on object perception in electric fish [ 21]. The echolocating bat provides a model system that reveals how precisely timed motor actions (vocal production) feed back to support spatial perception for orientation in a complex auditory scene.

The analysis of auditory scenes has been studied largely in human listeners who can report their perceived segregation and streaming of auditory objects under passive listening conditions [ 22]. The concept of auditory scene analysis, however, applies broadly to all hearing animals and extends to active listening situations. Auditory scene analysis by active listening, or echolocation, holds importance to unraveling general principles of spatial perception, because sonar targets are auditory objects, and the echolocating bat controls the features of its sonar vocalizations to extract information about the locations of these auditory objects in the environment. By studying the vocal production patterns of the echolocating bat as it negotiates a complex environment, we can directly measure its responses to a dynamic auditory scene. In this context, we have previously reported evidence that the bat's perceptual system can integrate acoustic information across echo streams, a requirement for tracking spatially distributed auditory objects (sonar targets) as it flies [ 2].

In the present study, we examined the FM bat's sonar signal production patterns as it pursued insect prey close to background vegetation and found that the big brown bat, *E. fuscus,* can successfully, but with apparent difficulty, take tethered insects positioned as close as 10 cm from the background clutter. Overall performance (success rate and trial time) improved with target–clutter separation, and with a separation of 40 cm the bat's behaviour was close to that observed in the open room. Past research on clutter rejection by echolocation in bats has emphasized measurement of the time-frequency structure of individual sonar cries [ 11, 13, 23– 25]. Bats foraging in the field receive a cascade of echoes following each sonar vocalization, and for this reason, we considered in this study the temporal patterns in sound sequences for the successful capture of insect prey. We predicted that when E. fuscus encountered prey positioned near vegetation it would adapt the temporal parameters of sound groups embedded in sonar sequences. In particular, we predicted an increase in strobing behavior as the bat approaches prey positioned close to clutter, a decrease in the interval of strobe group PIs during its approach to prey near clutter, and a decrease in the duration of the terminal buzz in the presence of clutter compared with open room. We also expected that the bat would adapt its flight path to enhance directional separation of the insect prey and background clutter.

The incidence of sonar strobing was higher in clutter than in the open room condition, and the increase in strobing occurred earliest with respect to target contact for the most challenging condition (20-cm clutter; see Figure 10A). The incidence of sonar strobing in the 40-cm clutter condition also increased over that in the open room condition, but this occurred later, in the 300–500 ms before target contact. This increase in strobing behavior is consistent with the notion that the sound groups may contribute to the bat's spatial representation of the foreground (target) and background (clutter) as well as their spatial separation. The stable PIs of strobe groups may also play a role in the bat's estimate of relative velocity, a parameter that would be important for controlling goal-directed flight and collision avoidance.

There were additional changes in temporal patterning of the bat's sonar cries under conditions of clutter; however, some were in the opposite direction from that which we predicted. Namely, bats foraging in the presence of clutter produced sonar strobe groups with longer time separations between individual sounds within the groups than under open room conditions. This pattern suggests that the clutter conditions led the bat to extend its listening/processing time following each vocalization in the strobe group ( Figure 10C).

The sounds within strobe groups were shorter in duration for the shortest target–clutter separation ( Figure 10E), a finding that did not emerge from analyses of the entire sound dataset (see Figure 9A). This finding is consistent with earlier reports on adaptive echolocation behavior in the presence of clutter, which have focused on the characteristics of individual sonar sounds [ 13]. In the present study, differences in signal duration between open room and 40-cm compared with the 20-cm clutter separation only appeared in the subset of sounds included in Figure 10E, signals contained within strobe groups during the time period 550 ms before target contact.

As described above, sonar orientation is an active system, involving the interplay between action and perception. The unexpected increase in sonar PIs within strobe groups under clutter conditions emphasizes the importance of perceptual processing time, which can be tightly coupled to the timing of motor behaviors. This is relevant to other spatially guided behaviors, such as collision avoidance (e.g., [ 26]). It is also important to note that even the “open room” condition in the lab compares to fairly cluttered conditions in the field, because of the limited size of the room. Field data suggest that under truly open conditions E. fuscus reduces PIs smoothly through the approach phase, whereas production of strobe groups is related to acoustic orientation in more cluttered habitats [ 10]. Thus, strobing behavior appears to be evoked by clutter conditions in which the bat must spatially track obstacles as well as targets, and the temporal characteristics of sonar strobe groups can be influenced by task demands.

The coupling of wingbeat, respiration, and vocal production contributes to the temporal patterning of sonar signals [ 18, 27– 29], and indeed this coupling may, in part, explain the sonar signal strobe groups we described here. More important, however, the timing of the strobe groups cannot be attributed strictly to the bat's respiratory/wingbeat cycle, which is consistent with Suthers et al.'s [ 18] original report on *Phyllostomus hastatus,* showing that pulses can be emitted at any point of the respiration cycle (hence corresponding wingbeat cycle). Our analyses show that the production of sonar strobe groups depends on the acoustic conditions the bat encounters, arguing against the notion that pulse rate is a simple by-product of the respiratory cycle. We have also examined the relationship between the timing of sonar strobe groups and the phase of wingbeat cycles, and the data show that sonar strobe groups occur at different phases of the wingbeat cycle ( Figure 6). Finally, the bat can vocalize continuously through a terminal buzz sequence over 300 ms in duration, which speaks to the flexible coupling between vocal production and respiration [ 2].

In response to background vegetation, the bat adapted the duration of the terminal buzz sound group, using shorter buzzes for the shorter target–clutter separations ( Figure 8). One might have predicted that buzz durations would be longer for the shorter target–clutter separations to provide extended time of rapid sampling of the target and background; however, the finding that buzz duration in fact shortens is consistent with our previous study of bat insect-capture behavior [ 2], as the bat appears to reduce the buzz duration to minimize the time during which it experiences mixing of pulses and the cascade of echoes returning not only from the prey but also from the branches of the clutter-producing plant.

The onset of buzz II can serve as an index of when the bat achieves precise 3-D localization of the target amid the background, which is required prior to its final attack on the prey [ 30]. Under more difficult conditions (shorter target–clutter separations), this happens later with respect to time-to-contact. Thus, the bat has less time to plan/control the final attack, which is corroborated by the later and more abrupt turns in flight paths seen under more difficult clutter conditions.

The time it took the bat to initiate insect capture in the presence of clutter changed with the target–clutter separation. At the closest separation tested (10 cm), the mean time to attempt insect capture was greater than 1 min, and this value dropped below 30 s for the 20-cm target–clutter separation and below 15 s for the 40-cm separation. In the field, the bat cannot take so much time to prepare for an attack, but the lab data demonstrate the limits of the bat's foraging behavior in proximity to clutter. Our results also suggest that a bat can find insects close to vegetation, and the motor control for fine maneuvering may limit its foraging behavior in dense foliage. In the field, the bat may detect insects close to clutter and exploit opportunities to intercept them when the prey increase their distance from vegetation, even by 10–20 cm. For the more challenging clutter conditions studied here in the laboratory, we hypothesize that the bat sampled spatial acoustic data over many tens of seconds to build a representation of the target and clutter before approaching the prey for interception and avoiding collision with the clutter.

We hypothesize that the sonar strobe groups produced by the bat during the approach phase are used to build a spatial representation of the environment from a collection of “snapshots,” and information extracted from these successive snapshots guides the update of appropriate motor behaviors. This is not to say that echoes from sonar cries that occur with changing PI do not also result in acoustic snapshots, as the duty cycle of FM bat signals is less than 10%, but we propose that the stable PI of strobe groups may support sharpening of the spatial images carried by the echoes and enable motor planning from echo information collected over time.

Neural recordings reveal sharpening of spatial response fields of auditory neurons in the bat midbrain when stimulated with simulated “strobe groups,” compared with variable PIs [ 15]. Of particular relevance to the bat's range-dependent adjustments in vocal production patterns is the dependence of echo-delay tuning on the temporal parameters of acoustic stimulation. Echo-delay tuning is characterized by an auditory neuron's facilitated and selective response to sound pairs within a restricted range of delays between a simulated sonar pulse and echo, and it is believed that this response property is involved in representing target distance information in the bat's sonar receiver [ 17, 31, 32]. It has been reported that the rate of fixed-interval sequences of pulse–echo pairs can affect the delay-tuning of auditory cortical neurons in the FM bat [ 17]. A more recent study reveals that the appropriate temporal patterning of sound sequences, approximating those produced by foraging bats, can uncover echo-delay tuning in auditory midbrain neurons, which otherwise respond weakly to acoustic stimulation [ 15]. These data suggest that the bat's active control over the temporal patterning of its vocalizations directly impacts the response profiles of auditory neurons that are involved in representing spatial information in the bat's environment.

## Materials and Methods

Five free-flying bats of the species E. fuscus were studied in these experiments. The bats were collected from attics of private homes in Maryland during the summers of 1999 and 2000. The animals were housed in a bat vivarium at the University of Maryland. The temperature of the vivarium was maintained at approximately 28 °C and the humidity at approximately 50%. Bats were exposed to a reversed light:dark cycle, with lights turned on in the animal colony room 14 h each day, between the hours of 6 pm and 8 am. Bats were maintained at approximately 80% of ad libitum feeding weights and allowed free access to water. All experiments were conducted between the months of June and September, when the bats are most active. One group of three bats was studied in the summers of 1999 and 2000, and another group of two animals was studied in the summers of 2001 and 2002.

### General behavioral methods

Experiments were carried out in a large flight room (7.3 m × 6.4 m × 2.5 m), lined with Sonex acoustic foam (Acoustical Solutions, Richmond, Virginia, United States) (see Figure 1). In all tasks, bats searched for insects under long wavelength (> 650 nm) lighting to eliminate the animal's use of vision [ 33]. Tethered mealworms were suspended at a height of about 1.5 m above the floor, and obstacles were placed at distances specified with reference to the edible targets.

### Video recordings

Two high-speed video cameras (240 Hz; MotionCorder, Eastman Kodak, Motion Analysis Systems Division, San Diego, California, United States) were positioned in the upper corners on one side of the flight room. A calibration frame (Peak Performance Technologies, Centennial, Colorado, United States) was placed in the room and filmed by both cameras prior to each recording session. The video cameras were used simultaneously to record positions of microphones, obstacles, target, and the bat's flight path. Each camera buffer could store a maximum of 8.2 s of video at 240 frames/s. Data were acquired continuously until the experimenter hit an end-trigger. The end-trigger controlled the video buffers of both cameras and was used to store the bat's flight behavior leading up to and following the pursuit, attack, and possible capture of tethered insect prey.

### Audio recordings

Full bandwidth echolocation signal recordings were taken by two microphones (Ultra Sound Advice, London, United Kingdom) placed on the floor within the calibrated space. The signals were then amplified (40 dB), band-pass filtered (10–100 kHz, Stanford Research Systems, Sunnyvale, California, United States), and recorded digitally on two separate channels of an IOtech WaveBook (sample rate 250 kHz/channel; IOtech, Cleveland, Ohio, United States). Sound acquisition was synchronized to the video system, and each stored sound file was 8.2 s in duration, corresponding to the length of the video buffer for a given trial.

### Video-processing methods

A commercial motion analysis system (Motus; Peak Performance Technologies) was used to digitize both camera views with a Miro DC30 Plus interface (Pinnacle Systems, Mountain View, California, United States). The Peak Motus system was also used to calculate the 3-D location of points marked in both camera views. Digitization was to 1/4-pixel resolution using magnification. The digitization procedure resulted in 2560 × 1920 lines of resolution. The video image spanned less than 6 m horizontally, so that 1/4-pixel resolution corresponded to approximately 0.4 mm. The accuracy of the system was within ±0.5% over a calibrated volume extending approximately 2.2 × 2.2 m across the room and 1.6 m vertically. The 3-D space calibration frame provided 25 control points for direct linear transformation calibration. The calibration procedure produced a mean residual error of 1.0 cm in each coordinate for the 25 control points.

The video position data for the bat, the target, and clutter-producing plants (when present) were entered in a database for each trial. The bat's position with respect to the microphones was also measured, and a correction factor for the sound travel time from the bat to the microphone was used to accurately record the vocalization times.

### Audio-processing methods

Recordings of the bat's sonar vocalizations in the laboratory insect-capture studies were processed in MATLAB. Data acquired digitally with the IOtech WaveBook were displayed as time waveforms and spectrograms (256 point FFT) using MATLAB (MathWorks, Natick, Massachusetts, United States). The onset time and duration of the signals were measured using the time waveforms, and frequency measurements were taken from the spectrograms. The onset time, duration, and start and end frequencies of the first harmonic of the emissions were marked with a cursor on the display and entered in a database for further analysis. Audio and video data were merged in a single analysis file in order to associate vocal behavior with motor events. MATLAB animation permitted dynamic playback of the bat's position data and corresponding vocalizations, enabling detailed study of the bat's behavior under different task conditions (programming by A. Schurger and A. Perez). Examples can be found at http://www.bsos.umd.edu/psyc/batlab/insect_capture_trials.

Sonar sound strobe groups were identified using the following criteria: two or more clustered sonar sounds that are bracketed by sounds occurring with a PI at least 1.2 times the mean PI of the clustered sound group. When three or more sounds occurred in a cluster, the PI of a strobe group was stable, with a tolerance of 5%. Note that the terminal buzz that occurs prior to insect capture contains a sequence of sonar sounds with a mean PI of less than 8 ms, and these signals were not included in the analysis of sonar strobe groups. The selection of these criteria developed out of detailed quantitative analyses of scores of trials; however, we note that it should be used as a working criterion, which may require modification for the analysis of vocalization data from other species and bats echolocating in different environments.

### Behavioral tasks

We studied the bat's performance in tasks that required the animal to use echolocation to detect, localize, and track a tethered insect positioned in proximity to vegetation (an artificial houseplant, resembling a fern, approximately 80 cm in diameter and 50 cm high, hanging from the ceiling at the same elevation as the tethered mealworm). Trials were run with the tethered insects at different distances from the vegetation, ranging from 10 cm to 40 cm ( *n* = 51 for 10 cm, *n* = 63 for 20 cm, and *n* = 70 for 40 cm). The plant was irregular in shape so distance was measured from the mealworm to the nearest part of the plant. The positions of the tethered insect and plant were changed from trial to trial, requiring the bat to continuously explore the environment to find its food reward. The bats were only fed during the behavioral trials and were therefore motivated to perform the task. The separation between the tethered insect and the plant was randomized from trial to trial, with an approximately equal number of trials run for each of three target–clutter separations on each test day. Control trials in which the bat caught tethered insects in an open room (without the plant) were also run ( *n* = 32). Trials in which the bat captured the prey or knocked it off the string were pooled. Both outcomes were the result of effectively localizing and contacting the prey. Previous results show that pre- and postcapture sonar signals are quite similar for target interception and hit trials, and these differ distinctly from signals produced by the bat when it aborts or misses completely [ 34].

During experiments, the bat was held in a small cage in the flight room and released at the start of each trial. The bat was released from a new direction each time. A stop watch was used by the investigator to record the duration of each trial, from the time of the bat's release to the time of the (attempted) insect capture. If a bat did not attempt to capture the tethered insect within 5 min of release, the trial was aborted, and the bat was returned to its cage for a 2-min timeout. If the bat made no attempt to take a tethered insect over three consecutive trials, the bat was returned to the colony room, and testing resumed the following day.

### Echo recordings

Echoes from the artificial houseplant (clutter object) and a tethered mealworm were measured. Sounds were generated using a Tucker-Davis System 2 (hardware and software; Tucker-Davis Technologies, Alachua, Florida, United States), amplified, filtered (Stanford Research Systems), and broadcast through a speaker (Tweeter LT800, frequency response flat [±3 dB] up to 100 kHz; Tweeter, Canton, Massachusetts, United States) powered with an Ultra Sound Advice DC amplifier. Echoes were picked up with a G.R.A.S. 1/4-inch microphone (type 40BF; G.R.A.S. Sound and Vibration, Holte, Denmark) amplified 40 dB by a Larson Davis (Provo, Utah, United States) amplifier/power supply, filtered by a Wavetech filter (band-pass 10–100 kHz; Karachi, Pakistan), and digitized online with a WaveBook (IOtech), using a sampling rate of 500 kHz. The emitted signal consisted of a 30-kHz tone shaped by a Hanning window function. The signal was 2 ms in duration and repeated every 150 ms. The speaker and the microphone were pointed from the same position toward the target, but the microphone was placed 10 cm closer to the target to maximize the signal-to-noise ratio. The speaker and microphone were placed 50 and 40 cm from the target, respectively. We measured the target strength of the clutter plant and mealworm at 30 kHz. Target strength calculations were referenced to 10 cm, and measured relative to the sound pressure level incident on the target.

## Abbreviations

3-D: three-dimensional
ANOVA: analysis of variance
FM: frequency-modulated
PI: pulse interval
